# A truncated form of CD9-partner 1 (CD9P-1), GS-168AT2, potently inhibits *in vivo* tumour-induced angiogenesis and tumour growth

**DOI:** 10.1038/bjc.2011.303

**Published:** 2011-08-23

**Authors:** S Colin, W Guilmain, E Creoff, C Schneider, C Steverlynck, M Bongaerts, E Legrand, J P Vannier, M Muraine, M Vasse, S Al-Mahmood

**Affiliations:** 1 Gene Signal Research Center, 4 Rue Pierre Fontaine, 91000 Evry, France; 2 Groupe de recherche MERCI (EA 3829), Faculté de Médecine and Pharmacie, 76000 Rouen, France; 3 Laboratoire SiRMa, UMR CNRS 6237, UFR Sciences de Reims, BP1039, 51687 Reims Cedex, France

**Keywords:** CD9P-1, GS-168AT2, angiogenesis, lung cancer, tetraspanin

## Abstract

**Background:**

Tetraspanins are transmembrane proteins known to contribute to angiogenesis. CD9 partner-1 (CD9P-1/EWI-F), a glycosylated type 1 transmembrane immunoglobulin, is a member of the tetraspanin web, but its role in angiogenesis remains to be elucidated.

**Methods:**

We measured the expression of CD9P-1 under angiogenic and angiostatic conditions, and the influence of its knockdown onto capillary structures formation by human endothelial cells (hECs). A truncated form of CDP-1, GS-168AT2, was produced and challenged *vs* hEC proliferation, migration and capillaries’ formation. Its association with CD9P-1, CD9, CD81 and CD151 and the expressions of these later at hEC surface were analysed. Finally, its effects onto *in vivo* tumour-induced angiogenesis and tumour growth were investigated.

**Results:**

Vascular endothelial growth factor (VEGF)-induced capillary tube-like formation was inhibited by tumour necrosis factor *α* and was associated with a rise in CD9P-1 mRNA expression (*P*<0.05); accordingly, knockdown of CD9P-1 inhibited VEGF-dependent *in vitro* angiogenesis. GS-168AT2 dose-dependently inhibited *in vitro* angiogenesis, hEC migration and proliferation (*P*<0.05). Co-precipitation experiments suggest that GS-168AT2 corresponds to the sequence by which CD9P-1 physiologically associates with CD81. GS-168AT2 induced the depletion of CD151, CD9 and CD9P-1 from hEC surface, correlating with GS-168AT2 degradation. Finally, *in vivo* injections of GS-168AT2 inhibited tumour-associated angiogenesis by 53.4±9.5% (*P*=0.03), and reduced tumour growth of Calu 6 tumour xenografts by 73.9±16.4% (*P*=0.007) without bodyweight loss.

**Conclusion:**

The truncated form of CD9P-1, GS-168AT2, potently inhibits angiogenesis and cell migration by at least the downregulation of CD151 and CD9, which provides the first evidences for the central role of CD9P-1 in tumour-associated angiogenesis and tumour growth.

Angiogenesis begins with the activation of endothelial cells and includes endothelial cell migration, proliferation and differentiation into capillaries (Carmeliet, 2000). Angiogenesis is essential for progression of solid tumours. Consequently, targeting angiogenesis has become a major focus in cancer drug development. Despite the initial enthusiasm for targeting angiogenesis for treatment of cancer, clinical trials, mainly based on vascular endothelial growth factor (VEGF) effect inhibition, have shown modest increases in survival. This can be due to the fact that angiogenesis is a complex multistep process of formation of new vessels that is regulated by numerous growth factors (Risau, 1997). Therefore, the inhibition of only one growth factor can lead to the overexpression of other different angiogenic factors, and it could be interesting to identify other targets which, in addition to the previous identified angiogenic growth factors, are involved in tumour angiogenesis.

Tetraspanins compose a family of proteins with four transmembrane domains delineating two extracellular domains of unequal size. These molecules have been implicated in numerous physiological processes including angiogenesis, cell migration, cell–cell contact and fusion (for reviews, see Boucheix and Rubinstein, 2001; Hemler, 2003; Levy and Shoham, 2005). Tetraspanins are also implicated in different diseases including tumour angiogenesis and metastasis (Boucheix and Rubinstein, 2001), hepatitis C virus and malaria sporozoites infections (Silvie *et al*, 2003; Cocquerel *et al*, 2006). The function of tetraspanins is thought to be related to their ability to interact with one another and with various other surface proteins, forming a network of molecular interactions referred to as the tetraspanin web. Inside the tetraspanin web, small primary complexes composed of particular tetraspanins associated with partner nontetraspanin proteins have been identified (Boucheix and Rubinstein, 2001). The demonstration that CD151 contributed to the interaction of the integrin *α*_3_*β*_1_ with other tetraspanins, as did CD9 for one of its molecular partners, CD9 partner-1 (CD9P-1), provided strong support for this scenario (Berditchevski *et al*, 2002; Charrin *et al*, 2003).

CD9 partner-1/FPRP/EWI-F is a glycosylated type 1 integral membrane protein (Orlicky and Nordeen, 1996). CD9 partner-1, a cell surface Ig superfamily protein, associates specifically with CD81 and CD9, but not with integrins (Stipp *et al*, 2001). CD9 partner-1 associates also CD151 but to a less extent (Charrin *et al*, 2001). Although CD81 associates with both *α*_3_ integrin and CD9P-1, it seems that the *α*_3_*β*_1_-CD81 and CD81-CD9-CD9P-1 complexes were distinct (Stipp *et al*, 2001). In spite of the evidences showing the associations of CD9P-1 with many tetraspanins, the role of CD9P-1 remain to be elucidated.

In this study, we show that CD9P-1 expression is essential for angiogenesis, and a truncated form of CD9P-1, GS-168AT2, inhibited dose-dependently human endothelial cell (hEC) proliferation, migration and *in vitro* and *in vivo* angiogenesis as well as the *in vivo* tumour growth, probably by downregulating of CD9 and CD151 at the cell surface.

## Materials and methods

### Animals, products and cell lines

Human umbilical vein endothelial cells and culture medium EGM-2MV was from Lonza (Levallois Perret, France), Bovine aortic endothelial cells (BAECs) were from American Type Culture Collection (ATCC, La Jolla, CA, USA). DMEM, RPMI-1640, fetal calf serum (FCS), IPTG (isopropyl-1-B-D-thio-1-galactopyranoside), kanamycine, chloramphenicol, calcium- and magnesium-free phosphate-buffered saline (PBS), trypsine-EDTA (Versene, Eurobio, Courtaboeuf, France), hypoxanthine aminopterine thymidine were from Eurobio. MTT (thiazolyl blue tetrazolium bromide), Tris pH 7.5, phenylmethylsulfonyl fluoride (PMSF), leupeptin, pepstatin A, aprotinin, detergent Brij 97 and Bradford reagent were from Sigma-Aldrich (Saint-Quentin Fallavier, France). Matrigel was purchased from Becton Dickinson (Le Pont De Claix Cedex, France). Bacteria culture medium LB, Thermoscript and the high fidelity Platinum HIFI enzymes were from Invitrogen (Cergy Pontoise, France). Qiaquick and Qiaprep miniprep were from Qiagen (Courtaboeuf, France), the RACE 5′3′ RACE kit was from Roche Applied Science (Meylan, France). Vascular endothelial growth factor and tumour necrosis factor *α* (TNF*α*) were purchased from R&D Systems (Lille, France). The pGEM-T easy vector, pCi neovector and antibiotic G418 were from Promega (Charbonnières-les-Bains, France). The pET30 vector, *E*. *coli* NovaBlue and *E. coli* BL21(DE3)pLys were from Novagen-Merck Biosciences (Nottingham, UK). The monoclonal antibody 229T anti-GS-168AT2 that recognises CD9P-1 was produced in our laboratory (Guilmain *et al*, 2011). Unless otherwise noted, antibodies were from Santa Cruz (Santa Cruz, CA, USA).

### Cell culture

Human EC were cultured in complete EGM-2MV medium as previously described (Al-Mahmood *et al*, 2009). Bovine aortic endothelial cells were grown in DMEM containing 10% FCS, and the transfected BAEC were maintained in DMEM, 10% FCS containing 300 *μ*g ml^–1^ of G418.

### Angiogenesis-related gene identification

The *in vitro* angiogenesis assay and the identification of angiogenesis-related genes were carried out according to methods described by our group (Al-Mahmood, 2000; Al-Mahmood *et al*, 2005). Briefly, hEC (second to sixth passages) were seeded onto type-1 collagen coated plates, and tested in three experimental conditions, that is, non-stimulated (control), stimulated with VEGF (50 ng ml^–1^, pro-angiogenic conditions), and the combination of VEGF (50 ng ml^–1^) and TNF*α* (50 ng ml^–1^) (angiogenesis inhibition conditions). This was followed by incubation at 37 °C under 5% CO_2_. At the maximum of capillary tube-like structure formation (48 h later with reload of factors at 24 h), cells were collected and RNA were extracted and analysed by differential display as previously described (Al-Mahmood, 2000).

### RNA preparation and analysis

After 48 h, the medium was aspirated and cells were lysed directly with lysis buffer (Qiagen). RNA were extracted with RNeasy Mini Kit (Qiagen) with Dnase treatment according to manufacturer's protocol. Reverse transcription and differential display were performed according Liang and Pardee (1992) except that two microgram of total ARN were reverse transcribed with superscript II (Invitrogen) following the manufacturer's protocol. Differential display PCR was performed with [*α*^33^P]dCTP (Amersham, Saclay, France) and PCR product (2 *μ*l) was analysed on 6% Genomyx HR-1000 acrylamide gels with Genomyx LR system (Beckman, Villepinte, France). Autoradiography was revealed after overnight exposition, bands of interest were cut and amplified with the same primers, cloned with pGEM easy vector system (Promega) following the manufacturer's protocol, sequenced analysed by questioning Blast program.

### CD9P-1 antisense preparation and stable cell line obtention

The PCR fragment identified by differential display was introduced in antisense orientation with respect to the PCI-neovector CMV constitutive promoter. The fragment of CD9P-1 gene was amplified using the forward primer (5′-CGG***GTCGAC***AGGTCCACTGCAGGGGGTTA-3′) in which a *Sal*I site was incorporated (italic letters) and the reverse primer (5′-CGC***ACGCGT***TTCCCCTTTGGAAGAGAGAGCA-3′) in which a *Mlu*I site (italic letters) was incorporated, generating a 392 pb fragment. The fragment and the vector were doubled digested with *Sal*I/*Mlu*I restriction enzyme (Promega). The fragment and the vector were ligated to each other. The plasmid containing this insert in the opposite orientation is propagated in *E. coli* JM109 (Promega) and purified with the Plasmid Mini Kit (Qiagen). The insertion in antisense orientation is controlled by sequencing, and the sequence of the inserted cDNA fragment (392 bp) corresponded to nucleotide number 4369 to nucleotide number 3978 (accession number: BC152454; gene id: 5738 PTGFRN). Bovine aortic endothelial cells were transfected with the plasmid (0.5 *μ*g DNA) using Effectene transfection reagent (Qiagen) according to the manufacturer's instructions. Briefly, BAEC were grown in DMEM (24-well plates) containing 10% FBS (Eurobio). At about 80% confluence, cells were washed with PBS and 1 ml of fresh medium and the complex (0.5 *μ*g DNA+60 *μ*l EC buffer+4 *μ*l enhancer+8 *μ*l effectene+350 *μ*l culture medium) were added. The cells were incubated for 6 h at 37 °C, 5% CO_2_, washed twice with PBS, and 1 ml of new medium was added and incubated overnight. Cells were collected by trypsinisation and plated at 8 × 10^4^ cells per well in presence of 700 *μ*g ml^–1^ of G418. The medium was changed every 3 days until complete elimination of cells in the control well (non-transfected cells). At confluence, cells were trypsined and maintained in the same medium containing 30 *μ*g ml^–1^ of G418. Stably transfected cells were obtained 3 weeks post-transfection in DMEM, 10% FBS (Eurobio) with 700 *μ*g ml^–1^ of G418 (Promega) and then maintained in the same medium containing 300 *μ*g ml^–1^ of G418.

### Cloning, production and purification of the recombinant protein GS-168AT2

The RNA library prepared from cells incubated with the combination of VEGF and TNF*α* were reverse transcribed into cDNA using Thermoscript enzyme; the resulting cDNA were then purified using the Qiaquick purification kit. The gene coding sequence was amplified using the high fidelity Platinum HIFI enzyme and the coupled primers 5′-GACGACGACAAGATGGCCTTTGATGTGTCCTGGTTTG-3′ and 5′-GAGGAGAAGCCCGGTTCAGGGATACTTGAAGGCGTTCAGCACA-3′ using the following program: 94 °C for 2 min; 35 cycles of 94 °C for 20 s, 60 °C for 20 s, 68 °C for 1 min and a final extension time of 7 min at 68 °C. The amplified DNA was purified by electrophoresis in agarose gel and inserted into pET-30 EK/LIC vector. For antisense transcript preparation, transfection and stable cell line generation, please see Supplementary Materials. The vector containing the insert was then amplified in *E*. *coli* NovaBlue, extracted, purified and validated by sequencing. The purified vector was used to transform *E. coli* BL21(DE3)pLys by heat shock (42 °C), and a positive colony for the presence of both the vector and the insert 168AT2 was grown in LB medium. The expression was induced by 1 mM IPTG, and the culture was furthered for 3 h. Cells were collected at 10^4^ × **g** for 10 min at 4 °C, and lysed with 25 U ml^–1^ of benzonase in 20 mM Tris-HCl buffer pH 8 containing 1 mM EDTA, 2 mM MgCl_2_, 1 mM PMSF, centrifuged at 10^4^ × **g** for 10 min at 4 °C, and the supernatant and the pellets were analysed by SDS–PAGE.

Bacteria cell lysates were centrifuged, and the insoluble fraction was collected with buffer A (20 mM Tris-HCl, pH 8.0, 8 M urea, 0.5 M NaCl, 5 mM imidazol) containing 5 mM GSH. The suspension was centrifuged, supernatants were collected, filtered onto 0.45 *μ*m membrane, and used to purify GS-168AT2 using a His-Trap column (Amersham) coupled to HPLC (Amersham). The sample was loaded onto the column equilibrated with buffer A, followed by extensive washing with buffer A followed by: (i) elution with a 6–4 M urea linear gradient in buffer A and (ii) a two steps elution with 0.3 and 0.5 M imidazol in buffer A containing 4 M urea. The recovered fraction was then subjected to two dialysis at 4 °C: (i) overnight against 20 mM Tris-HCl, pH 8, 150 mM NaCl, 4 M urea; and (ii) for 3 h against buffer B (20 mM Tris-HCl, pH 8, 50 mM NaCl, 2 M urea, 0.1 mM CaCl_2_). As at high concentrations (>1.2 mg ml^–1^) GS-168AT2 tends to precipitate, it was stored under solution in buffer B. The purified protein was then filtered through 0.45 *μ*m membranes and protein content was quantified by Bradford assay and by SDS–PAGE coupled to gel slab analysis using Gene Genius system (Syngene, Cambridge, UK).

We have also cloned, produced and purified another recombinant protein corresponding to a truncated form of the cell surface tetraspanin 7/TM4SF2 (gene accession number: emb∣CAB65594.1; gene ID: 7102 TSPAN7) (amino acid no. 176–218). The purified recombinant protein has a molecular mass of 16 kDa and is used as a negative control protein (NCP) in animal experiments.

### General procedures

For proliferation assay, cells were cultured in the presence of 10 *μ*l of either 2 M urea in 0.9% saline (vehicle) or increasing concentrations of GS-168AT2 in vehicle for 42 h. Cell proliferation was measured by the MTT assay (Mosmann, 1983) using *μ*Quant micro-plate reader coupled to the KC4 software (BioTek Instruments GMBH, Colmar, France).

The *in vitro* angiogenesis assay, cell labelling, angiogenesis quantification and IC_50_ calculations were performed as previously described (Al-Mahmood *et al*, 2009). Cell migration was tested by the wound assay (Sato and Rifkin, 1988). Briefly, confluent cell monolayer was scraped with a plastic tip on one line and the culture medium was renewed with medium supplemented with either GS-168AT2 or vehicle as control. After 18±1 h of incubation, plates were placed on the stage of an inverted microscope (Olympus, Rungis, France) and each well was photographed. To quantify *in vitro* internalisation of GS-168AT2, cells were taken in *Laemmli* buffer, spun at 10^4^ × **g** for 15 min, and supernatants were resolved by SDS–PAGE and immunoblotted with the anti-GS-168AT2 mAb (229T mAb) or anti-CD9 mAb (clone H110).

### Cell treatment and immunoprecipitations

All antibodies used in these and other immunoprecipitation experiments have been previously shown to immunoprecipitate their target antigens (Sheikh-Hamad *et al*, 2000; Stipp *et al*, 2001; Clarck *et al*, 2004). Human EC grown in EGM-2MV (80% confluence) were incubated with GS-168AT2 (40 *μ*g ml^–1^) or vehicle for the indicated time, washed three times in cold PBS and directly lysed in 2 ml of ice-cold lysis buffer (10 mM Tris, pH 7.5, 150 mM NaCl, 1 mM PMSF, 0.5 *μ*g ml^–1^ leupeptin, 1 *μ*g ml^–1^ pepstatin A and 1 *μ*g ml^–1^ aprotinin) containing 1% detergent Brij 97, 1 mM CaCl2 and 1 mM MgCl2, by incubation for 30 min at 4 °C. Cell lysates were spun at 10^4^ **g** for 10 min, insoluble materials were discarded, and protein contents of supernatants were measured by Bradford assay and adjusted at similar concentrations. Cell lysates (1 ml) were precleared with 25 *μ*l of protein G-plus agarose beads (Santa Cruz) for 30 min, and proteins were then immunoprecipitated by adding 2 *μ*g of anti-CD9 (clone ALB 6), anti-CD81 (clone 5A6) or anti-CD151 (clone 11G5a) mAbs for 1 h, the immunocomplexes were pulled down with protein G-plus agarose beads and the beads were washed three times with lysis buffer. The immunoprecipitates were separated by NuPAGE 4–12% Bis-Tris gel electrophoresis under reducing conditions, transferred to PVDF membrane (Novex System, Invitrogen), and the membrane was blocked with 5% (w/v) non-fat milk in TBS containing 0.1% v/v Tween-20 for 1 h. The membrane was incubated with the indicated primary antibody for 2 h, washed three times and incubated with the appropriate HRP-conjugated secondary antibody and revealed by enhanced chemiluminescence, ECL plus (GE Healthcare, Velizy, France).

### Flow cytometry

Following incubation, cells were collected using nonenzymatic cell dissociation solution (Sigma; Saint-Quentin Fallavier, France), washed with cold PBS and incubated (10^6^ cells) for 15 min at 4 °C with 5 *μ*l of phycoerythrin-anti-CD9 mAb conjugate (25 *μ*g ml^–1^, clone M-L13, Becton Dickinson). Cells were washed twice with PBS, and directly analysed for CD9 (clone ALB 6), CD81 (clone 5A6) or CD151 (clone 11G5a) staining by flow cytometry (EPICS XL-MCL, Coulter). Data were expressed by subtracting the background fluorescence produced by the negative control antibody (Isotypic IgG1, clone MOPC-31C, BD Biosciences, Le Pont-De-Claix Cedex, France) from the specific antibody.

### Tumour-induced *in vivo* angiogenesis

All experiments with animals were reviewed by the Genopole's institutional animal care and use committee and were performed in accordance with institutional guidelines for animal care. Female BALB/c nu/nu mice (*n*=10) were from Charles Rivers (St Germain sur l’arbresle, France). The human non-small cell lung carcinoma (NSCLC) cell lines Calu-6 cell line was obtained from ATCC were grown in RPMI containing 10% FCS at 37 °C and 5% CO_2_ humidified atmosphere. For each plug, tumour cells (5 × 10^6^ cells in 50 *μ*l of HBSS) were added to 350 *μ*l of Matrigel (Becton Dickinson), and the mixture was subcutaneously injected into the right flanks of the mice. After 24 h, mice were randomised and separated into two groups of five mice each. All treatments were started at day 1 post inoculation, and realised by intraperitoneal (i.p.) injection with a fixed volume of 200 *μ*l per injection. Control mice (group 1) were daily injected with vehicle for 8 days. Group 2 was daily treated (eight injections) with GS-168AT2 dissolved in vehicle at 1 mg ml^–1^. At the end of treatments (day 8), animals were anaesthetised, and plugs were harvested, weighed and photographed. Haemoglobin content was spectrophotometry measured at 540 nm using Drabkin Reagent Kit 525 (Sigma-Aldrich) and used for the quantification of blood vessels. Absorbance results were compared against a standard curve of haemoglobin. Haemoglobin content was expressed as g/g of wet matrigel. Data were analysed using two-tailed Student's *t*-test. Results showing *P*-values <0.05 were considered as significant.

### Tumour xenografts in nude mice and GS-168AT2 administration

Female BALB/c nu/nu mice (*n*=25) were used at 5–6 weeks of age. The animals were housed in laminar air-flow cabinets under pathogen-free conditions with a 14-h light/10-h dark schedule, and fed by autoclaved standard chow and had water *ad libitum*. Calu-6 (5 × 10^6^ cells in 200 *μ*l of serum-free RPMI) were subcutaneously injected at the right flank of the mice. After engraftment (10 days), tumour volume (TV) was measured (Balsari *et al*, 2004), and animals were randomised, and separated into five groups, five animals each, to be treated by i.p. injection (200 *μ*l per injection) every other day for 16 days (eight injections). Control mice (group 1) received the vehicle (2 M urea in 0.9% saline). Cis-diammine platinium II dichloride (CDDP, Sigma) was dissolved in 0.9% saline at 0.5 mg ml^–1^, and injected at a dose of 5 mg kg^–1^ (group 2).

Negative control protein was dissolved in vehicle at 1.5 mg ml^–1^ and injected at a dose of 15 mg kg^–1^ (group 3). GS-168AT2 was dissolved in vehicle at 1.5 mg ml and injected at a dose of 15 mg kg^–1^ (group 4). In group 5, mice received both CDDP and GS-168AT2. Tumour volume and body weight were measured every other day over the treatment period (16 days) by two independent scientists and data were statistically analysed using two-tailed Student's *t*-test.

### Statistic

All data were analysed with Prism 5 (GraphPad Software Inc., La Jolla, CA, USA) using two-tailed Student's *t*-test. All the data were presented as mean±s.e., where *n* is the number of independent experimentations. *P*-value was considered as significant when <0.05.

## Results

### The essential role of CD9P-1 during angiogenesis

**Figure 1:**
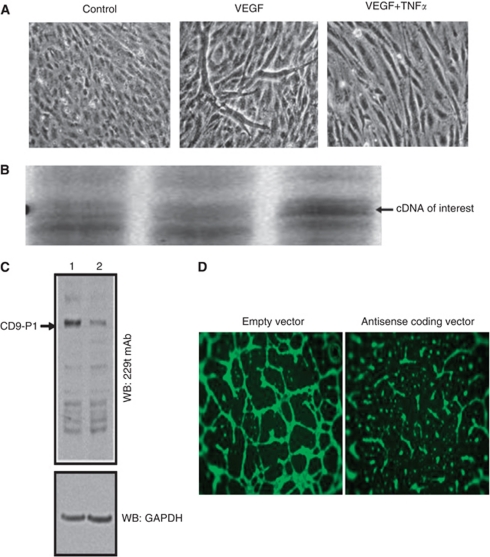
Angiogenesis modulation by CD9P-1 expression. (**A**) Human EC were incubated with culture medium alone (control), or supplemented with either VEGF (50 ng ml^–1^), or TNF*α* at antiangiogenic concentration (50 ng ml^–1^), for 48 h with reload of factors at 24 h. (**B**) Gene profiling of the differentially regulated genes in the above conditions. The cDNA of interest marked with an arrow, was identified by sequencing as the CD9-P1 gene. (**C**) Representative image of WB of BAEC harbouring the empty (lane 1) or CD9-P1 antisense transcript coding pci-neovector (lane 2) using 229T mAb. (**D**) Representative images of *in vitro* angiogenesis assay with BAEC harbouring the empty or CD9P-1 antisense transcript coding pci-neovector.

Exposure to VEGF (50 ng ml^–1^) led to differentiated hEC-derived tube-like structures (*in vitro* angiogenesis); this effect of VEGF was prevented by TNF*α* (50 ng ml^–1^) (Figure 1A). Differential gene expression profiling revealed that, compared with controls, the overexpression of CD9P-1 mRNA (identified by sequencing) was detected in the presence of TNF*α* (Figure 1B).

We cloned the identified cDNA fragment in the antisense orientation in a pCI neovector, which was used to transform BAEC; in these conditions, EC harbouring the vector coding for CD9P-1-specific antisense transcript expressed much less CD9P-1 protein relative to EC harbouring empty vector (Figure 1C). EC harbouring the vector coding for CD9P-1-specific antisense transcript formed 70±12% less tube-like structures (*P*<0.01; *n*=6) than EC harbouring the empty vector (Figure 1D), revealing that the expression of CD9P-1 is a regulator for EC to undergo *in vitro* angiogenesis.

### A truncated form of CD9P-1, GS-168AT2, inhibits dose-dependently *in vitro* angiogenesis, hEC migration and proliferation

**Figure 2:**
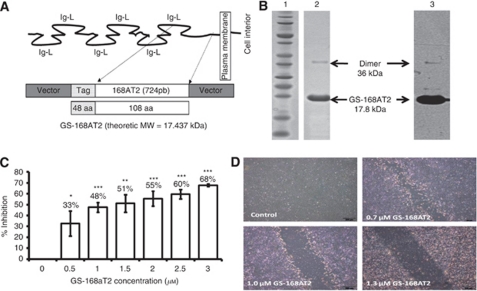
The truncated form of CD9-P1, GS-168AT2, inhibits dose-dependently *in vitro* hEC proliferation and migration. (**A**) Schematic representation of the structure of CD9-P1, the cloned truncated CD9-P1 domain, GS-168AT2, and the produced Tag-recombinant protein. (**B**) SDS–PAGE imprints (lane 1, molecular mass marker; lane 2 purified GS-168AT2) and the corresponding WB (lane 3, using 229 mAb) of the purified GS-168AT2 used for the all subsequent experiments. (**C**) Dose-response curve of the effects of GS168AT2 on the proliferation of hEC. Results were expressed as percentage of control±s.e. (*n*=4). Statistical significance between the control and the different doses of GS-168AT2 were calculated with two-tailed Student's *t*-test (^*^P<0.05; ^**^P<0.01; ^***^P<0.001). (**D**) Representative images at 18 h of the wounded hEC monolayer incubated with either vehicle or increasing concentrations of GS-168AT2.

To investigate the importance of CD9P-1-induced expression, we cloned and produced a truncated form of CD9P-1 named GS-168AT2 (amino acid 724–832), corresponding to the extracellular portion close to the transmembrane domain of CD9P-1 (Figure 2A). The choice of the produced truncated form was based on the fact that this sequence corresponds to the unglycosylated part of CD9P-1, and it is the most adjacent to the plasma membrane; and thus could potentially be the region by which CD9P-1 laterally interacts with other cell surface partners. GS-168AT2 was extracted and purified to homogeneity (Figure 2B). Of interest, GS-168AT2, under its purified state, dimerised to some extent as revealed by the presence of a 36 kDa band using Comassie blue (Figure 2B, lane 2) and the same band was recognised by WB with the 229T mAb rose against it (Figure 2B, lane 3). For all the subsequent experiments, we have used GS-168AT2 as a solution in buffer B (which contained 2 M urea, please see Materials and Methods section) while the vehicle was buffer B alone.

**Figure 3:**
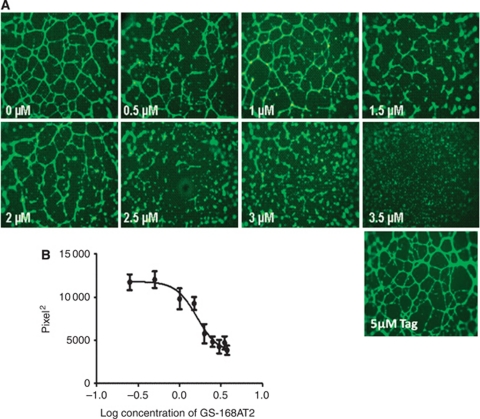
The truncated form of CD9P-1, GS-168AT2, inhibits dose-dependently *in vitro* angiogenesis. (**A**) Representative images showing that GS-168AT2 inhibits *in vitro* angiogenesis in a concentration-dependent manner, and (**B**) its quantification. Results were expressed as mean±s.e. (*n*=4).

GS-168AT2 inhibited hEC proliferation with an IC_50_ of 2.78±0.46 *μ*M (*n*=4) (Figure 2C) and modestly the proliferation of Calu-6 (12.8±4.9% and 38.6±3.9% inhibition at 2.5 and 5 *μ*M GS-168AT2, respectively; *n*=4). In contrast, GS-168AT2 had no significant effect on the proliferation of the hamster ovarian cancer cells (CHO cells), MRC5 (human fibroblast cell line) or the myelomonocytic cell line U937 (data not shown). GS-168AT2 also inhibited hEC migration with a threshold concentration of 0.7 *μ*M and a maximal inhibitory effect at 1.35 *μ*M (*n*=4) (Figure 2D). Finally, GS-168AT2 inhibited in a dose-dependent manner *in vitro* angiogenesis with an IC_50_ of 1.75±0.13 *μ*M (*n*=4) (Figure 3A and B). Neither vehicle, nor 5 *μ*M N-terminal Tag influenced *in vitro* angiogenesis (Figure 3A).

### GS-168AT2 co-precipitates with both CD9 and CD151, and poorly with CD81

**Figure 4:**
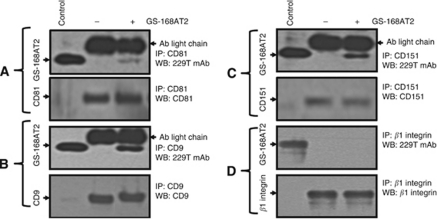
GS-168AT2 co-precipitates with both CD9 and CD15, but poorly with CD81. Human EC were incubated with vehicle or GS-168AT2 for 2 h, washed twice with vehicle and hEC were lysed in 1% Brij 97 lysis buffer, and the indicated tetraspanins were immunoprecipitated (CD9, CD81 and CD151) by incubating the cell lysate (the protein contents of cell lysates were adjusted by Bradford protein assay) with the indicated antibody at 4 °C. The immunocomplexes were pulled down with agarose beads coated with G-protein, and the beads were washed twice with lysis buffer. The immunoprecipitates were resolved in SDS–PAGE and western blotted with the indicated antibody. GS-168AT2 was used as control. (**A**) Upper panel: WB with 229T mAb of the immunoprecipitates with anti-CD81 mAb showing that GS-168AT2 poorly co-precipitated with CD81. Lower panel: WB with anti-CD81 of the immunoprecipitated CD81 showing equivalent quantities of CD8 deposited in the upper panel. (**B**) Upper panel: WB with 229T mAb of the immunoprecipitates with anti-CD9 mAb showing that GS-168AT2 co-precipitated with CD9. Lower panel: WB with anti-CD9 of the immunoprecipitated CD9 showing equivalent quantities of CD9 deposited in the upper panel. (**C**) Upper panel: WB with 229T mAb of the immunoprecipitates with anti-CD151 mAb showing GS-168AT2 co-precipitated with CD151. Lower panel: WB with anti-CD151 of the immunoprecipitated CD151 showing equivalent quantities of CD151 deposited in the upper panel. (**D**) Upper panel: WB with 229T mAb of the immunoprecipitates with anti-*β*1 mAb showing GS-168AT2 do not co-precipitated with *β*1 integrins. Lower panel: WB with anti-*β*1 mAb of the immunoprecipitated integrin *β*1 showing equivalent quantities of integrin *β*1 deposited in the upper panel. Western blots presented in this figure are representative images of at least three independent experiments.

In the aim to identify potential partners at hEC susceptible to interact with GS-168AT2, hEC were incubated with GS-168AT2 followed by cells washes and immunoprecipitation of CD9, CD81 and CD151 with their respective antibodies. Immunoblotting with 229T mAb raised against GS-168AT2 showed there was a modest quantity of GS-168AT2 pulled down with CD81 (Figure 4A). In contrast, there were more important quantities of GS-168AT2 co-precipitated with CD9 and CD151 (Figure 4B and C), indicating that GS-168AT2 directly or indirectly interacts with both CD9 and CD151. In contrast, no interaction with *β*1 integrins were observed (Figure 4D).

### Degradation of GS-168AT2 is associated with depletion of both CD9 and CD151 from cell surface

**Figure 5:**
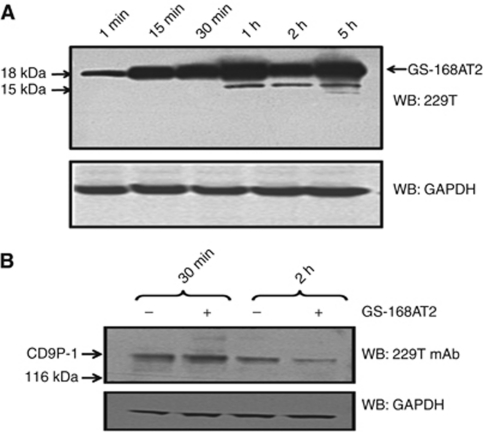
Proteolysis of GS-168AT2 by hEC is associated with an important downregulation of CD9P-1 at the cell surface. Human EC were incubated with GS-168AT2, and collected at the indicated time, washed twice with vehicle, lysed and lysates resolved by SDS–PAGE. (**A**) The kinetic of GS-168AT2 degradation by hEC was monitored by WB of cell lysates with the 229T mAb and compared with GAPDH as an internal standard. Arrows indicate the intact GS-168AT2 and the proteolytic fragments (15 kDa) of GS-168AT2 recognised by the 299T mAb. (**B**) The downregulation of CD9P-1 was monitored by WB of cell lysates with the 299T mAb and compared with GAPDH as an internal standard. Western blots presented in this figure are representative images of at least three independent experiments.

GS-168AT2 was incubated with hEC, followed by cell washes and analysis of the fate of GS-168AT2 over time. Results showed that there were important quantities of intact GS-168AT2 throughout the experimental time; this was associated with apparition of a degraded form (15 kDa) with time (Figure 5A).

The fate of CD9P-1 was assessed. WB with the 229T mAb showed that there were decreasing quantities of CD9P-1 with time when hEC were incubated with GS-168AT2 (Figure 5B), suggesting the downregulation of CD9P-1, either alone or in association with other partners(s).

**Figure 6:**
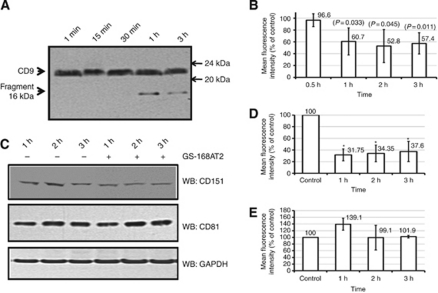
GS-168AT2 induced depletion of both CD9 and CD151 from cell surface. Human EC were incubated with either vehicle or GS-168AT2, and collected at the indicated time and washed twice with vehicle. Cell were then either lysed and resolved by SDS–PAGE, or directly analysed for the amount of CD9, CD151 and CD81 at the cell surface by FACS using the respective antibodies of these tetraspanins. All the results of FACS analysis were presented as the mean fluorescence intensity relative to control (hEC incubated with vehicle) and represent the mean±s.e. of four separate experiments realised in triplicate. Statistical significance were calculated with two-tailed Student's *t*-test (^*^P<0.05). (**A**) WB of the cell lysate with an anti-CD9 mAb monitoring relatively stable amounts of CD9 and the apparition of proteolytic fragment (16 kDa) of CD9 recognised by the anti-CD9 mAb with time in the presence of GS-168AT2. (**B**) Analysis of the hEC by FACS using anti-CD9 mAb showing that GS-168AT2 induced time-dependent decrease in CD9 at the cell surface. (**C**) WB of the cell lysate with anti-CD151 or CD81 mAbs monitoring decreased amounts of CD151 with time in hEC incubated with GS-168AT2 relative to vehicle, while there was not significant change in the level of CD81. (**D**) Analysis of the hEC by FACS using anti-CD151 mAb showing that GS-168AT2 induced time-dependent decrease in CD151 at the cell surface. (**E**) Analysis of the hEC by FACS using anti-CD81 mAb showing that GS-168AT2 did not induced significant changes in the level of CD81 at the cell surface. Western blots presented in this figure are representative images of at least three independent experiments.

The status of CD9, CD81 and CD151 with time was also assessed. Following exposure of hEC to GS-168AT2, cells were lysed, and cell lysates were western blotted with an anti-CD9 mAb. Results indicated that there were no significant variations in the amounts of CD9 (21 kDa) in the presence of GS-168AT2 with time (Figure 6A), probably due to the important pool of intracellular CD9 (Kovalenko *et al*, 2004). However, there was the apparition of a 16 kDa fragment detectable from 1 h of incubation of hEC with GS-168AT2 (Figure 6A), which could correspond to a fragment of CD9 as it was recognised by the anti-CD9 mAb. To investigate further, the status of CD9 at the cell surface, hEC incubated with GS-168AT2 were also analysed by FACS. Results showed that there were important decreases (about 40% less; *P*<0.05; *n*=4) in the amount of CD9 at the cell surface with time in the presence of GS-168AT2 relative to control (Figure 6B).

WB of cell lysate with anti-CD151 mAb showed there was also a time-dependent decrease in CD151 in the presence of GS-168AT2 (Figure 6C). This was further confirmed by the analysis of hEC by FACS, which showed that the amount of CD151 at the cell surface was significantly decreased (69.66±10.99% decrease; *P*<0.01; *n*=4) relative to hEC exposed to vehicle (Figure 6D). Interestingly, the kinetic of both CD151 (Figure 6C and D) and CD9 (Figure 6B) downregulation correlated with each other and both correlated with that of GS-168AT2 degradation showed in Figure 5A. Investigation of CD81 status, however, showed any significant changes following cells exposure to GS-168AT2 (Figure 6C and E).

### GS-168AT2 inhibits the *in vivo* tumour-induced angiogenesis and tumour growth

**Figure 7:**
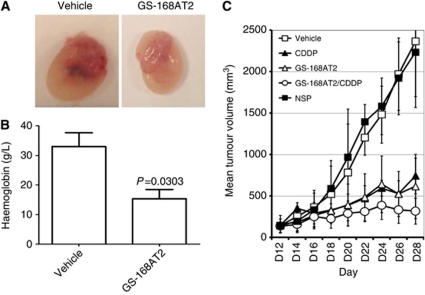
GS-168AT2 inhibits the *in vivo* angiogenesis and tumour growth. (**A**) Representative images of tumour-enriched plugs treated with either vehicle or GS-168AT2. (**B**) Haemoglobin contents of tumour-enriched plugs treated with either vehicle or GS-168AT2. (**C**) Mean tumour volume curve of mice bearing Calu-6 tumours and treated with vehicle, nonspecific protein (NCP) at 15 mg kg^–1^, CDDP at 5 mg kg^–1^, GS-168AT2 at 15.0 mg kg^–1^, or combined GS 168A-T2 at 15.0 mg kg^–1^ and CDDP at 5 mg kg^–1^. For both haemoglobin dosing in (**B**) and TV measurements on (**C**), results were expressed as means±s.e. of *n*=5, and were considered statistically significant when *P*-value <0.05 using two-tailed Student's *t*-test.

We first examined the influence of GS-168AT2 onto the *in vivo* angiogenesis using tumour-enriched Matrigel plugs model. Before treatments, there were no significant variations in the volume of tumour-enriched Matrigel plugs subcutaneously implanted in Nude mice. At the ends of treatments, while plugs issued from vehicle-treated group have a red colour, plugs issued from GS-168AT2-treated group have a white-yellowish colour with sporadic small red spots suggesting that GS-GS-168T2 inhibits tumour-induced neovascularisation *in vivo* (Figure 7A).

Quantification of haemoglobin in plugs as a marker of neovascularisation showed that plugs issued from mice treated with GS-168AT2 had 53.4±9.5% (*n*=5; *P*=0.03) less haemoglobin contents than plugs issued from vehicle-treated mice (Figure 7B) suggesting that GS-168AT2 potently inhibited *in vivo* tumour-induced angiogenesis.

All nude mice bearing Calu-6 tumours survived during the therapy. Before therapy, there were no significant differences for nude mice in weight and TVs. The mean TV (MTV) at day 28 in mice of group 2 treated with NCP was similar to that of mice treated with the vehicle (group 1), and there were no significant differences between the two groups (*P*>0.05) (Figure 7C). The MTV at day 28 in mice of group 3 treated with CDDP (742.4±215.8 mm^3^) was significantly (*P*=0.008) smaller than MTV of vehicle-treated mice (2364.6±663.8 mm^3^) (Figure 7C) indicating that CDDP provoked 68.6±9.1% inhibition of *in vivo* tumour growth. The MTV at day 28 was decreased (*P*=0.008) in group 4 treated with GS-168AT2 alone (615.9±388.5 mm^3^) suggesting that GS-168AT2 provoked 73.9±16.4% inhibition of the *in vivo* tumour growth. The first statistical significance between group 1 and the other treated groups was observed at day 8 post-treatment for groups 3, 4 and 5 (Figure 7C). An important reduction of the MTV (364.2±105.6 mm^3^) was observed in animals from group 5 treated with the combination of CDDP and GS-168AT2 compared with group 1 (*P*=0.008) (Figure 7C), indicating that the combined treatment with CDDP and GS-168AT2 provoked 86.6±6.6% of *in vivo* tumour growth.

Unlike GS-168AT2, which had no impact on mouse body weight (24.20±1.37 g) compared with vehicle-treated mice (24.40±1.19 g), CDDP led to a loss in body weight (*P*<0.05) both when administered alone (18.69±1.97 g) or in combination with GS-168AT2 (18.39±0.74 g) (Table 1).

## Discussion

Tetraspanins associate with themselves or with other membrane proteins to form microdomains, called the tetraspanin web. Some previous studies have suggested that tetraspanins could have a role in angiogenesis (Zhang *et al*, 2009). In this study, we show that the level of expression of a partner of tetraspanins is crucial for *in vitro* angiogenesis, and the truncated form of CD9P-1, the GS-168AT2, potently inhibits *in vitro* and *in vivo* angiogenesis, possibly by associating both CD9 and CD151 leading to their depletion from hEC surface.

CD9P-1 associates CD9, CD81 and to a lesser extent CD151 (Charrin *et al*, 2001; Stipp *et al*, 2001), yet, the precise role of CD9P-1 remains to be elucidated. Structurally, CD9P-1 protein has a large extracellular domain with many glycosylated Ig-like domains, and an unglycosylated hydrophobic plasma membrane-adjacent domain that we have produced and named GS-168AT2. Our data showed clearly that GS-168AT2 inhibited dose-dependently hEC proliferation, migration and *in vitro* capillary tube formation, suggesting multi-inhibition mechanisms. The antiangiogenic activity of GS-168A is important, as it is at least 300- and 600-fold more potent than the VEGFr (KDR) and Tie 2 receptor-derived peptides in matter of concentration, respectively (Binétruy-Tournaire *et al*, 2000; Tournaire *et al*, 2004).

Consistently with earlier data (Stipp *et al*, 2001; Guilmain *et al*, 2011) showing the interaction of CD9P-1 with CD9 and CD151, our data showed that GS-168AT2 co-precipitated with both CD9 and CD151 suggesting a direct and/or an indirect interaction of GS-168AT2 with these tetraspanins. The possibility of co-precipitation of GS-168AT2 with CD9 and CD151 was due to the interaction of GS-168AT2 with endogenous CD9P-1 could not be ruled out. Indeed; (i) GS-168AT2 dimerises under its purified state (this study), suggesting that GS-168AT2 under its monomer state could dimerise with its homologous sequence within CD9P-1; (ii) GS-168AT2 co-precipitated with CD9P-1 (Guilmain *et al*, 2011) and (iii) CD9P-1 dimerises in tetraspanin-independent and dependent manners at the cell surface of many cell types (André *et al*, 2009). A limitation to this hypothesis is the lack of demonstration that GS-168AT2 inhibits CD9P-1 dimerisation and/or oligomerisation. Nonetheless, our results also showed that GS-168AT2 poorly co-precipitated with CD81suggesting at least that GS-168AT2 does not correspond to the amino acid sequence of CD9P-1, which associates CD81.

Our data showed that the co-precipitation of GS-168AT2 with both CD9 and CD151 was accompanied by important depletions of CD151 and CD9 from the cell surface, most probably by their internalisation and degradation. Additionally, CD9 and CD151 depletion courses matched with the downregulation course of CD9P-1, and all of these were correlated with the degradation of GS-168AT2 (most probably following its internalisation) which could suggest the internalisation of the molecular complex. Nonetheless, the depletion of CD9, CD151 and CD9P-1 from hEC surface by GS-168AT2 could underlay its anti-migratory and antiangiogenic activities. Indeed, anti-CD9 antibody impaired EC migration (Klein-Soyer *et al*, 2000; Deissler *et al*, 2007), the expression of CD151 was correlated with angiogenesis and EC motility (Yáñez-Mó *et al*, 1998), and anti-CD151 antibody or CD151-knockdown lead to impaired angiogenesis and EC motility (Zhang *et al*, 2002; Takeda *et al*, 2007). Furthermore, CD9P-1 overexpression in HEK-293 cells increased cell migration (Chambrion and Le Naour, 2010), and we have recently shown that CD9P-1 expression localises at the migratory edges of human lung tumours, and positively correlates with their metastatic status (Guilmain *et al*, 2010). Our results presented in this study extend these findings and demonstrate GS-168AT2 dose-dependently inhibits hEC migration and the *in vitro* and *in vivo* angiogenesis.

It is widely established that tumour angiogenesis correlates with the metastatic status of tumours and tumour growth. Our results show that GS-168AT2 potently inhibits the *in vivo* tumour-induced angiogenesis in nude mice. Additionally, treatment of Calu-6-xenografted mice with GS-168AT2 (15 mg kg^–1^) alone for 16 days reduced tumour growth by about 73.9±16.4% (*P*=0.008). This effect was similar to that of treatment with CDDP alone, and the combined treatment of GS-168AT2 and CDDP led to 86.6±6.6% inhibition of tumour growth. These data confirmed our recent results obtained with another human NSCLC cell line showing that GS-168AT2 potently inhibited the *in vivo* growth of NCI-H460 (Guilmain *et al*, 2010). Additionally, the fact that NCI-H460 cells have wild-type p53 (Mitsudomi *et al*, 1992), while the Calu-6 cells harbour mutated p53 (Lehman *et al*, 1991), demonstrate that the in vivo growth inhibition of the two human NSCLC cell lines by GS-168AT2 is independent of the p53 genotype and it is most probably due to the antiangiogenic activity of GS-168AT2. This illustrates the greater efficacy of bitherapies (chemotherapy associated with antiangiogenic therapy) at reducing hLT proliferation. The antiangiogenic activity of GS-168AT2 most probably accounts for most of its antitumour growth effect as GS-168AT2 has modest influence onto Calu-6 growth *in vitro*. The efficacy of GS-168AT2 could be the sum of its direct effect on tumour cells and its antiangiogenic activity.

Our data suggest that the level of CD9P-1 expression is critical for the stability of CD9 and CD151 at hEC surface, and consequently for hEC migration, proliferation and angiogenesis and confirm that the ratio of expression levels between CD9P-1 and its tetraspanin partners can regulate cell motility (Chambrion and Le Naour, 2010). Excess (hEC stimulation with TNF*α*, similarly to the exogenous addition of GS-168AT2) or poor (experimentation with antisense) expression of CD9P-1, both lead to angiogenesis impairments. In agreement, our data suggest that the level of CD9P-1 expression is a crucial and limiting factor for CD9 and CD151 assembly at the surface of endothelial cells. This hypothesis clearly deserves further investigations.

In conclusion, our results show for the first time that CD9P-1 expression is essential for angiogenesis, and the truncated form of CD9P-1, GS-168AT2 impaired *in vitro* and *in vivo* angiogenesis by downregulating CD151 and CD9. Further knowledge on the role of CD9P-1 in both angiogenesis and tumour growth may reveal this pathway as a new and safe therapeutic target in oncology.

## Figures and Tables

**Table 1:** Mean body weight of mice bearing Calu-6 tumours and treated with vehicle, CDDP at 5 mg kg^–1^, GS-168AT2 at 15 mg kg^–1^, and combined GS 168A-T2 at 15.0 mg kg^–1^ and CDDP at 5 mg kg^–1^

	**Day post-treatment**				
**Treatment**	**Start of treatment**	**Day 4**	**Day 8**	**Day 12**	**Day 16**
Vehicle	22.35±1.17 g	24.08±1.23 g	24.17±1.17 g	23.59±1.14 g	24.40±1.19 g
CDDP	21.40±0.85 g	22.19±1.04 g	21.06±1.41 g	19.46±1.49 g	18.69±1.97 g
GS-168A-T2	21.74±0.89 g	22.78±0.99 g	23.28±0.90 g	22.90±0.59 g	24.20±1.37 g
GS-168A-T2/CDDP	20.74±1.41 g	21.29±0.59 g	20.13±0.78 g	19.76±1.16 g	18.39±0.74 g

Abbreviation: CDDP=cis-diammine platinium II dichloride.

Results were expressed as mean±s.e. (*n*=5).
