# Supplementary material for: A truncated form of CD9-partner 1 (CD9P-1), GS-168AT2, potently inhibits in vivo tumour-induced angiogenesis and tumour growth
Source: Br J Cancer. 2011 Aug 23;105(7):1002–11. doi: 10.1038/bjc.2011.303 (PMC3185932; doi:10.1038/bjc.2011.303)
Supplement: Supplementary Information [file bjc2011303x1.doc]

**Supplemental data**

**CD9P-1 antisense preparation and stable cell line obtention**

The PCR fragment identified by differential display was introduced in antisense orientation with respect to the PCI-neovector CMV constitutive promoter. The fragment of CD9P-1 gene was amplified using the forward primer (cgg***gtcgac***aggtccactgcagggggtta ) in which a SalI site was incorporated and the reverse primer (cgc***acgcgt***ttcccctttggaagagagagca) in which a MluI site was incorporated generating a 392 pb fragment. The fragment and the vector were doubled digested with SalI/MluI restriction enzyme (Promega). The fragment and the vector were ligated to each other. The plasmid containing this insert in the opposite orientation is propagated in *E. Coli* JM109 ( Promega) and then purified with a Plasmid Mini Kit (Qiagen). The insertion in antisense orientation is controlled by sequencing (Fig. S1). BAEC cells were transfected with the plasmid (0.5 µg DNA) using Effectene transfection reagent (Qiagen) according the manufacturer instructions. Briefly, BAEC cells were grown in DMEM (24 wells-plates) containing 10% FBS (Eurobio). At about 80% confluence, cells were washed with PBS and 1 ml of new medium and the complex (0.5 µg DNA+ 60 µL EC buffer + 4 µL Enhancer + 8 µL effectene + 350 µL culture medium) were added. The cells were incubated for 6 hr at 37°C, 5% CO2, washed twice with PBS, and 1 mL of new medium was added and incubated overnight. Cells were collected by trypsinization and plated at 80,000 cells/well in presence of 700 µg/mL of G418. The medium is changed every three days until complete elimination of cells in the control well (non transfected cells). At confluence, cells were trypsined and maintained in the same medium containing 30 µg/mL of G418. Stably transfected cells were obtained three weeks post-transfection in DMEM, 10% FBS (Eurobio) with 700 µg/mL of G418 (Promega) and then maintained in the same medium containing 300 µg/mL of G418.

TTCCCCTTTGGAAGAGAGAGCAGTGTTAGATCCTTCAAAGTCAAGATAAA

AGCAGAGGATTGCAATTAGGGCATGAGTCACCAGCGAGCCTCAGAAAACC

CTACTGGGATGCTTCCGAAAGGCCAGTGCTGTGCAGGGGGCGAGACACTT

CAGGGAGCGGGAGGCTCCAAGGACAGAGCTTTTAAAGCAGCAGCAGAAGC

CTGGGATGTGGAAGATCTCTGTCTACTCTCCTATTCCTGATGGGAGCAGT

CGGGCAAAGCACACCGCTCTTGACTCTGCTCTACAGGCCATTCACATAAC

AGCAAATTGCTGGGTTTCCTTCATGTCCCTATTAGGAAAGCAAAACATAC

CACAAAGTATACCCTTTCTCACTAACCCCCTGCAGTGGACCT

Figure S1: The sequence of the cDNA fragment inserted in the antisense orientation in the PCI-neovector CMV constitutive promoter.

**Inhibition of CD9P-1 expression by siRNA**

Human umbilical vein endothelial cell were cultured in 6-wells plate with complete EGM-2MV medium as previously described (Al-Mahmood *et al*., 2009). At about 80% of confluence, culture medium was removed, and cell layers were washed three times with PBS. Cell layers were then incubated with a mixture composed of the tranfection agent, N-Ter peptide (Sigma, ref N2788-120UL) , and 10 µM of either non specific siRNA as negative control (siRNAc) (Sigma, ref. VC300A2 and VC300B2); or with CD9P-1 specific siRNA, sense: 5' [AmC6F] CGAAGUACAUCAUCUCUCU [DT] and antisense: 5'[phos]A[2-5 RNZ G]AGAGAUGAUGUACUUCG [dt] [dT] (siRNA CD9P-1); or with buffer (Control). Following 2 hr of incubation at 37°C, EGM2 culture medium was added and the incubation was furthered for 48 hr. Cells were there then washed with PBS, and lyzed. Cell lysates were resolved in SDS-PAGE and immunoblotted with either 229T mAb or anti-actin mAb.


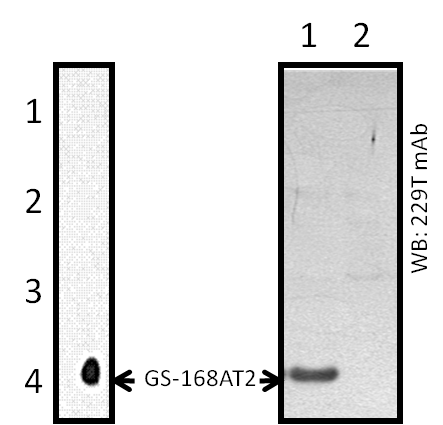

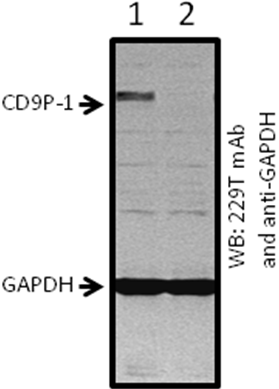


A

B

C


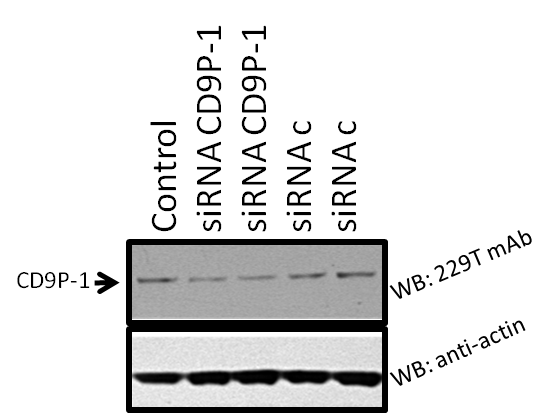


D

Figure S2: Characterization of 229T mAb. A) Vehicle (1) and two His-Tag recombinant proteins developed in our laboratory (2, 3) or GS-168AT2 (1) were immobilized on nitrocellulose membrane, immunoblotted with 229T mAb, and revealed with anti-mouse-HRP antibody. Only GS-168AT2 was recognized by 229T mAb. B) Purified GS-168AT2 (lane 1) or vehicle (lane 2) was resolved by SDS-PAGE, and western blotted with 229T mAb. Lane 1 presents a band of 18 kDa corresponding to GS-168AT2. C) Cell lysates of either BAEC (lane 1), or BAEC stably expression pci-neovector coding for CD9P-1 antisense transcript (lane 2) were resolved by SDS-PAGE, and successively western blotted with 229T and anti-GAPDH mAbs. Lane 1 presents two bands of about 135 and 36 kDa corresponding to CD9P-1 and GAPDH respectively. Lane 2 presents the band of 36 kDa corresponding to GAPDH, but not the band of 135 kDa corresponding to CD9P-1 indicating the inhibitions of the expression of CD9P-1 by the antisense transcript. D) Human umbilical vein endothelial cell was incubated with either non specific siRNA as negative control (siRNAc) or with CD9P-1 specific siRNA (siRNA CD9P-1), or with buffer (Control). Cells were lyzed and cell lysates were resolved in SDS-PAGE and western blotted with either 229T mAb or anti-actin mAb. Results indicate the CD9P-1 specific siRNA (siRNA CD9P-1) inhibits the expression of CD9P-1 and the 229 mAb monitored this inhibition of expression.
